# A Deep Learning Quantification Algorithm for HER2 Scoring of Gastric Cancer

**DOI:** 10.3389/fnins.2022.877229

**Published:** 2022-05-30

**Authors:** Zixin Han, Junlin Lan, Tao Wang, Ziwei Hu, Yuxiu Huang, Yanglin Deng, Hejun Zhang, Jianchao Wang, Musheng Chen, Haiyan Jiang, Ren-Guey Lee, Qinquan Gao, Ming Du, Tong Tong, Gang Chen

**Affiliations:** ^1^College of Physics and Information Engineering, Fuzhou University, Fuzhou, China; ^2^Fujian Key Lab of Medical Instrumentation & Pharmaceutical Technology, Fuzhou University, Fuzhou, China; ^3^Department of Pathology, Fujian Cancer Hospital, Fujian Medical University Cancer Hospital, Fuzhou, China; ^4^College of Electrical Engineering and Automation, Fuzhou University, Fuzhou, China; ^5^Department of Electronic Engineering, National Taipei University of Technology, Taipei, Taiwan; ^6^Imperial Vision Technology, Fuzhou, China; ^7^Fujian Provincial Key Laboratory of Translational Cancer Medicin, Fuzhou, China

**Keywords:** CNN, deep learning, gastric cancer, HER2 score prediction, re-parameterization

## Abstract

Gastric cancer is the third most common cause of cancer-related death in the world. Human epidermal growth factor receptor 2 (HER2) positive is an important subtype of gastric cancer, which can provide significant diagnostic information for gastric cancer pathologists. However, pathologists usually use a semi-quantitative assessment method to assign HER2 scores for gastric cancer by repeatedly comparing hematoxylin and eosin (H&E) whole slide images (WSIs) with their HER2 immunohistochemical WSIs one by one under the microscope. It is a repetitive, tedious, and highly subjective process. Additionally, WSIs have billions of pixels in an image, which poses computational challenges to Computer-Aided Diagnosis (CAD) systems. This study proposed a deep learning algorithm for HER2 quantification evaluation of gastric cancer. Different from other studies that use convolutional neural networks for extracting feature maps or pre-processing on WSIs, we proposed a novel automatic HER2 scoring framework in this study. In order to accelerate the computational process, we proposed to use the re-parameterization scheme to separate the training model from the deployment model, which significantly speedup the inference process. To the best of our knowledge, this is the first study to provide a deep learning quantification algorithm for HER2 scoring of gastric cancer to assist the pathologist's diagnosis. Experiment results have demonstrated the effectiveness of our proposed method with an accuracy of 0.94 for the HER2 scoring prediction.

## 1. Introduction

China is a high incidence area of gastric cancer, accounting for 46% of new gastric cancer cases in the world (Alatab et al., 2020). Human epidermal growth factor receptor 2 (HER2) positive gastric cancer is an important subtype of gastric cancer, and immunotherapy targeting HER2 significantly improves the prognosis of patients with advanced gastric cancer, which has become the first-line standard of care for advanced gastric cancer (Qiu, 2016). The Trastuzumab for Gastric Cancer (ToGA) study clarified that proper detection and evaluation of HER2 protein expression and gene amplification status of gastric cancer are of great significance for the clinical diagnosis and treatment of gastric cancer (Bang et al., 2010). However, the HER2 positive rate of gastric cancer in China is only 12–13% (Qiu, 2016).

Current evaluation of HER2 protein expression on HER2 immunohistochemically (IHC) stained sections is mainly performed manually, and the IHC method are still the preferred method for HER2 in gastric cancer (Li et al., 2016). As Table 1 shows, pathologists usually use a semi-quantitative assessment method to assign HER2 scores for gastric cancer by repeatedly comparing hematoxylin and eosin (H&E) whole slide images (WSIs) with their HER2 IHC WSIs one by one under the microscope, and continuously switching the microscopic field of view magnification to find suspicious cancerous areas, which are classified as 0, 1+, 2+, and 3+ according to the percentage of tumor cell membrane staining and staining intensity score. IHC3+ cases are directly determined as HER2 positive. IHC1+ and IHC0 cases are directly determined as HER2 negative. IHC2+ cases are “equivocal” cases, needing further evaluation by fluorescence *in situ* hybridization (FISH) to finalize HER2 status. If there is amplification, they are classified as HER2 positive, otherwise, the results are negative. In clinical practice, there are differences in the positive rate between different hospitals. Furthermore, the inefficient diagnosis can be due to the error prone and subjectivity of visual judgment. In China, many laboratories in primary care hospitals are not equipped for HER2 testing, which results in some HER2 positive patients not receiving timely and effective targeted therapy.

**Table 1:** Immunohistochemistry scoring guidelines for interpretation of Human epidermal growth factor receptor 2 (HER2) protein expression in gastroesophageal junction adenocarcinoma.

**Resection specimen staining pattern**	**Score**	**Classification**
No reactivity or membranous reactivity in <10% of cells	0	Negative
Faint/barely perceptible membranous reactivity in >10% of tumor cells	1+	Negative
Weak/moderate complete or basolateral membranous reactivity in >10% of tumor cells	2+	Equivocal
Moderate/strong complete or basolateral membranous reactivity in >10% of tumor cells	3+	Positive

With the gradual maturation of deep learning, Artificial Intelligence (AI) has been used in various fields such as agriculture, military, and transportation. Combining deep learning with clinical work has also become a research hotspot in recent years. In addition, intelligent diagnosis based on image data and treatment prognosis has been successfully applied in computed tomography (CT), magnetic resonance imaging (MRI), and X-ray imaging. In recent years, as image acquisition has been significantly improved, AI-assisted diagnosis of pathological images has also gradually become a research hotspot. The notable results include the identification of tumor regions such as skin (Andre et al., 2019), gastrointestinal (Song et al., 2020), and lung (Coudray et al., 2018), especially breast cancer pathological images have been extensively studied (Zhou et al., 2020). Compared to breast pathological images, there are few studies related to gastric cancer (Ai et al., 2021), especially the HER2 score evaluation of gastric cancer on IHC images. Additionally, WSIs have billions of pixels in an image, which poses computational challenges on Computer-Aided Diagnosis (CAD) systems. The previously proposed deep learning network framework requires a large number of computational resources as the neural network deepens, resulting in a major expenditure of time in processing images, which is not practicable for hospital diagnosis.

In this study, we proposed a novel automatic HER2 scoring framework, which can quantify HER2 assessment metrics and predict HER2 scores to assist physicians in clinical diagnosis. Our proposed method consists of two parts, the Tile-level classification network (TLCN) and the WSI-level HER2 score prediction network (WHSPN). Overall, our proposed framework can rate HER2 scores quantitatively, and serve as a reference for clinical diagnosis to reduce subjective differences in semi-quantitative ratings by pathologists.

In summary, the contributions of this study are 3-fold as follows:

1. We proposed a novel HER2 automatic scoring framework in gastric tumors that enables HER2 classification on patches, followed by the predictions of HER2 scores on WSIs.
2. To avoid segmentation and manual intervention, an automatic classification algorithm is proposed, which largely reduces the complexity of the analysis. Our framework introduces re-parameterization, which separates the training model from the deployment model. In addition, our proposed model can reduce computational costs while achieving similar performance and better meeting the hospital's equipment requirements.
3. Our approach only uses the HER2 stained slides to achieve quantification of HER2 score, reducing subjective variation in semi-quantitative interpretation by pathologists and achieving an accuracy of 94% on WSIs.

The remainder of this article is structured as follows. Section 2 briefly summarizes the study related to gastric histopathology image analysis, HER2 interpretation, and re-parameterization. Section 3 describes the methods. Experimental results and Discussions are presented in Sections 4 and 5.

## 2. Related Works

### 2.1. Gastric Histopathology Image Analysis

Gastric cancer is a common and fatal disease. Due to the scarcity of pathologists, CAD systems have been gradually introduced to assist pathologists in interpretation, and GHIA has become a hot research topic in recent years. Korkmaz and Binol (2018) used traditional machine learning methods such as ANN, RF, LBP, and HOG to detect early gastric cancer. Liu et al. (2018b) proposed an SVM classifier instead of a nonlinear classifier for the classification of gastric slices, and the method achieved 95% classification accuracy. Garcia et al. (2017) designed a nine-layer DCNN to automatically detect lymphocytes on gastric cancer IHC images. Nan et al. (2017) proposed a reiterative learning framework for partial labeled gastric tumor segmentation. Li et al. (2018) designed two modules, a shallow multiscale and a deep network, for better feature extraction, and evaluated the classification of this network based on IHC images of the public BOT gastric section dataset, which could achieve 97.93% classification accuracy. Liu et al. (2018a) constructed a 50-layer residual network to automatically detect tumor regions in IHC sections of gastric cancer. Wang et al. (2019) designed a recalibrated multi-instance deep learning method to solve the gastric cancer WSI classification puzzle. Sun et al. (2019) used deformable convolution and multi-scale embedding networks for accurate gastric cancer segmentation. Zhu et al. (2020) combined channel attention modules with spatial attention modules to construct a weakly-supervised balanced attention network for gastric pathology image localization and classification. Kosaraju et al. (2020) simultaneously extracted target regions at both high magnification and low magnification levels for accuracy analysis and validated its feasibility on well-differentiated, moderately-differentiated, and poorly-differentiated gastric adenocarcinoma tissues. Iizuka et al. (2020) used Inception-V3 as the basic network framework to classify WSI into adenocarcinoma, adenoma, and non-neoplastic. Sun et al. (2020) proposed a method for segmentation of gastric tumor regions based on a hierarchical conditional random field.

### 2.2. HER2 Pathology Images

Due to the prognostic importance of HER2 scoring, the highly subjective nature of the pathologists' diagnosis and the limitations of fatigue, CAD systems have been gradually introduced for HER2 interpretation. In breast cancer, the primary method for quantitative and semi-quantitative assessing HER2 IHC stained response consists of feature extraction followed by traditional machine learning (Ellis et al., 2005; Masmoudi et al., 2009). Brügmann et al. (2012) developed software to assess the IHC stained response of HER2 based on cell membrane connectivity 2012. Although commercial algorithms have been allowed to aid HER2 interpretation, commercial software is not only expensive but also requires manual intervention. The automated HER2 scoring contest (Contest, 2016) held in Nottingham in 2016 demonstrated that automated IHC scoring algorithms can provide quantitative HER2 assessment of morphological features and the automated methods can beat the pathologists on their data set. With the development of deep learning, the feasibility of HER2 interpretation has also been greatly improved. Vandenberghe et al. (2017) have validated the feasibility of deep learning in assisting HER2 interpretation with 83% agreement. However, this method relies on the accurate annotation of various types of cells. Singh and Mukundan (2018) verified the feasibility by learning texture features and intensity features of the input images with different saturation levels and using three architectures for them. Saha and Chakraborty (2018) proposed a deep learning network containing trapezoidal long short-term memory named HER2Net to implement HER2 scoring. Both of these works only realized HER2 classification on patches, lacking the predictions of HER2 scores on whole slides. Khameneh et al. (2019) achieved cell membrane segmentation by fine-tuning U-Net for HER2 status assessment. However, its complex cell membrane labeling is a huge challenge for pathologists. Qaiser and Rajpoot (2019) proposed a new IHC scoring attention model to focus on regions of interest in WSIs through deep reinforcement learning. Cordeiro et al. (2018) studied the implementation of HER2 interpretation using color features, and they used a traditional machine learning approach for feature extraction and classification. However, the method leads to a major expenditure of time on feature extraction. Zhang et al. (2020) proposed a HER2 scoring system that can be integrated into an augmented reality microscope. Their system can provide results to the pathologists while reading the slide. However, manual annotation of nuclei and membranes in these methods is challenging work. Compared to breast cancer, HER2 scoring in gastric cancer is mainly in FISH images (Zakrzewski et al., 2019; Schmell et al., 2020). Zakrzewski et al. (2019) developed a detection network called RetinaNet to detect HER2 amplification status based on FISH images. Schmell et al. (2020) added a VGG-like Nucleus Classifier network (Zakrzewski et al., 2019) to achieve FISH-based HER2 oncogene amplification testing. However, the cost of FISH testing is high, and only 2+ cases in the hospital require further evaluation by FISH. Sharma et al. (2017) explored a deep learning approach on H&E stained histopathology, but did not achieve HER2 scoring and can only achieve superficial cancer classification (cancer and normal) with an accuracy of 0.6990. The staining pattern of the HER2 IHC section in gastric cancer was not as clear as that in breast cancer. Therefore, the study of digital pathological sections of gastric cancer is still limited to superficial segmentation and classification (cancer and normal), and HER2 interpretation has not been studied in depth to assist clinical treatment. Although some progress has been made in the use of CAD systems to assist HER2 interpretation of breast cancer, segmentation and manual intervention are required. These methods not only rely on large-scale manual annotation, but also need a lot of computing resources for their complex analysis methods.

### 2.3. Re-parameterization

To accelerate CNNs for efficient inference, many methods have been proposed. For instance, Hinton et al. (2015) proposed a knowledge distillation method, which learns useful information from the knowledge learned by a large model to train a small model and performs model compression with similar performance. Although knowledge distillation can effectively improve the inference speed of the model, the training process is complicated. A teacher network and a student network need to be trained, and the degree of teacher network training will directly affect the student network. In deep learning, it is customary to use the training network as the deployment network like knowledge distillation, and re-parameterization is to convert the training network with a large number of parameters into a deployment network with a small number of parameters through parameter fusion, and the deployment network can greatly reduce parameters compared with the training network, but the accuracy is almost not degraded. Unlike knowledge distillation, re-parameterization only requires training a network and then converting it to the corresponding deployment network, which will greatly reduce the training complexity. In recent years, reparameterization has been applied in object detection, image segmentation, and image classification (Ding et al., 2019, 2021a,b). Zhang et al. (2021) designed a very efficient super-resolution network using re-parameterization. As far as we are concerned, the re-parameterization module has not been applied to the medical field.

## 3. The Proposed Method

**Figure 1:**
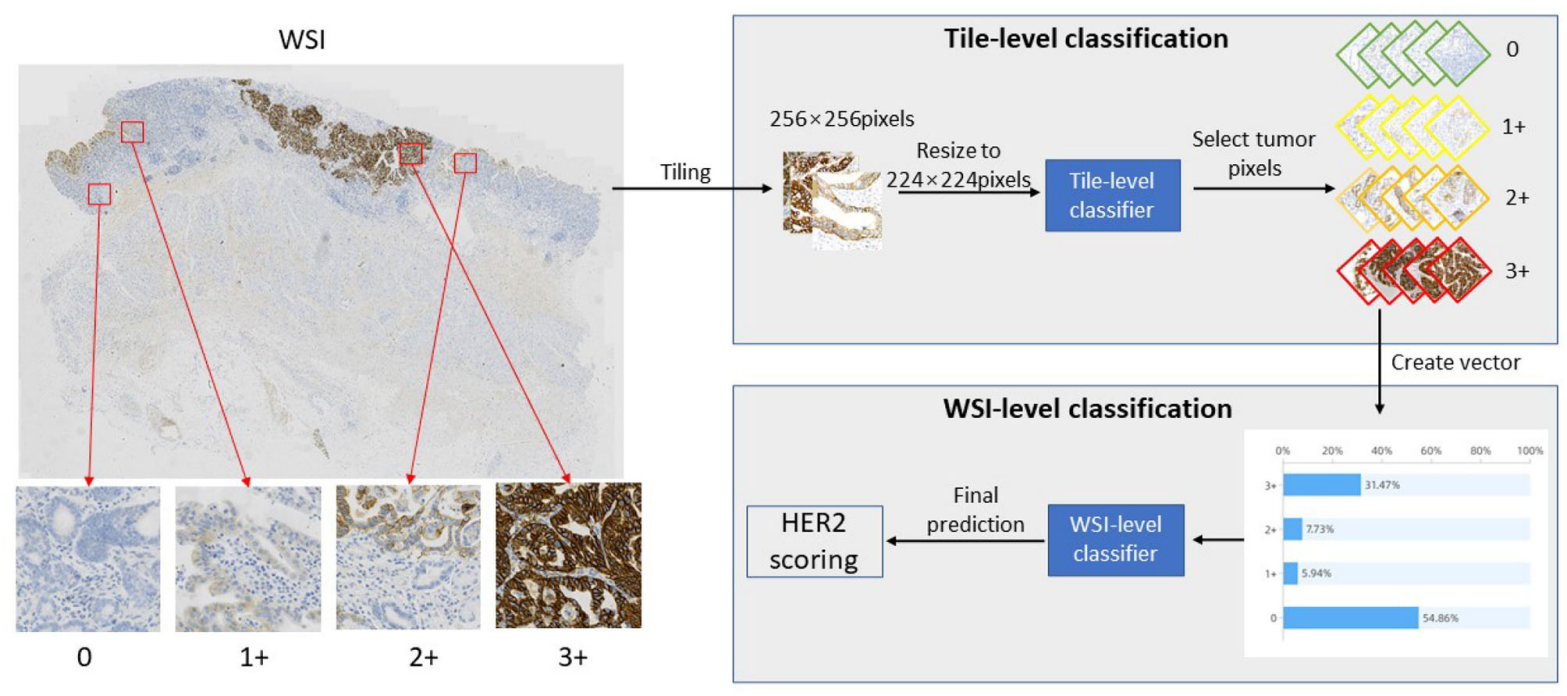
The Whole Framework of Quantification Algorithm for HER2 scoring of gastric cancer.

In this study, we proposed a novel automatic HER2 scoring framework, which can quantify HER2 assessment metrics and predict HER2 scores to provide pathologists with clinical assistance. This framework consists of two parts, the first part is the TLCN, which is able to obtain the categories of each patch image, where the re-parameterization is introduced to accelerate the computational process of intelligent interpretation. The second part is the WHSPN, which builds a vector based on the categories obtained from the TLCN and develops the HER2 scoring rules to predict the HER2 score in WSI-level autonomously. The whole framework of the quantification algorithm for HER2 scoring of gastric cancer is shown in Figure 1.

As shown in Figure 1, first, a slide is cropped into a patch-level images collection by a sliding window, and then this image collection is fed into the TLCN to predict the category of each patch image. Meanwhile, the percentage distribution of 0, 1+, 2+, and 3+ categories in the large WSI tumor region is counted, and these four category percentages are used to construct a one-dimensional vector. Finally, the one-dimensional vector is put into the WHSPN to obtain the final HER interpretation of the WSI.

### 3.1. Tile-Level Classification Network

The purpose of this step is to make the network learn the characteristics of relevant image patches. In this step, the extracted image patches with a size of 256 * 256 pixels are inputs. The outputs include six (0, 1+, 2+, 3+, normal, and noise) classes and a concatenated vector of the probability of 0, 1 +, 2 +, 3+ patches in tumor cells patches. The label of each patch used in the TLCN model was selected out of WSIs with six (0, 1+, 2+, 3+, normal, and noise) classes by the pathologist.

**Figure 2:**
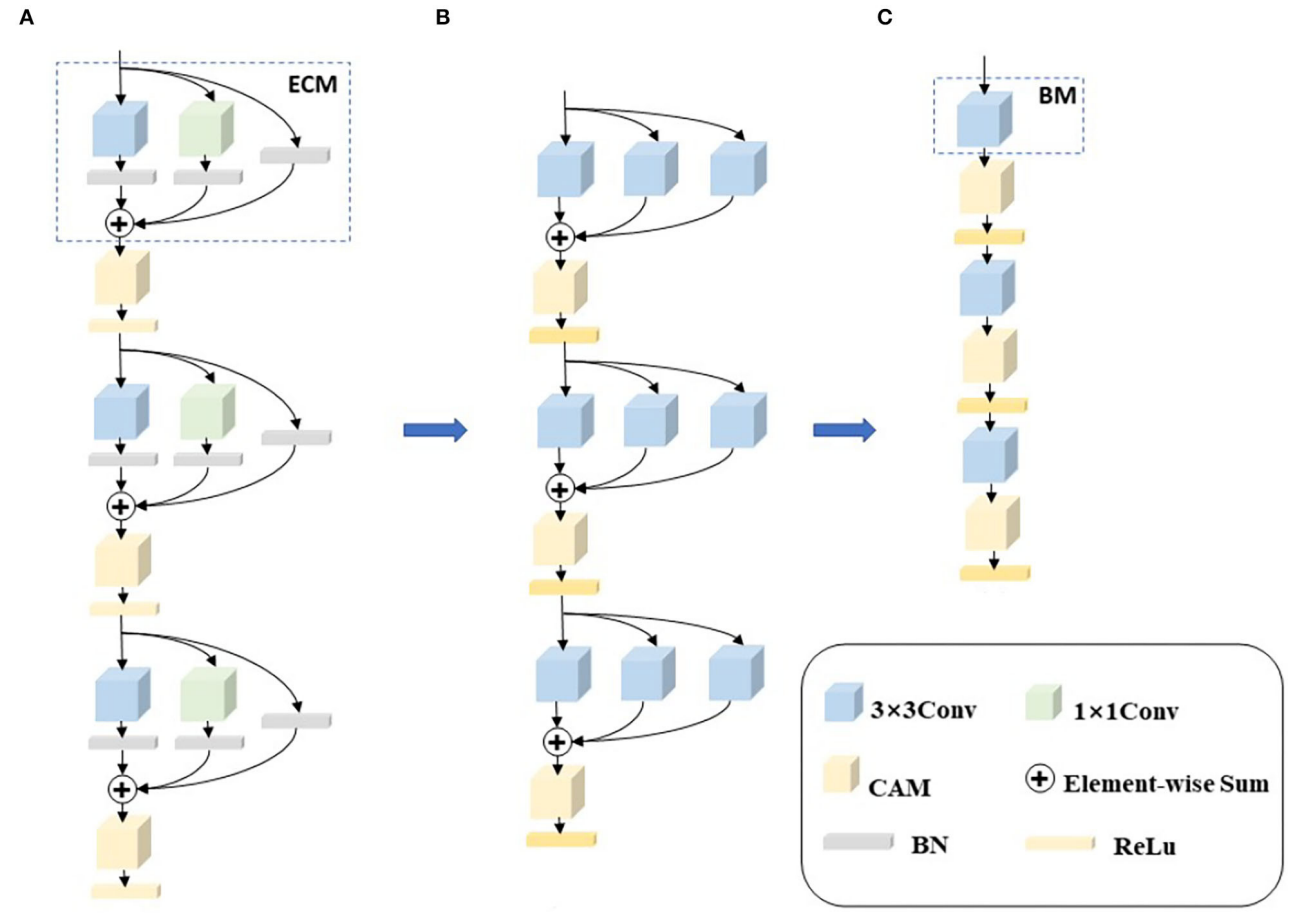
The whole framework of re-parameterization. **(A)** Is a training network composed of enhanced revolution modules (ECM). **(B)** Is the re-parameterization process 1×1 convolution and skip connections are converted to 3×3 convolutions, and then the three convolutions are combined into one convolution through element-wise adding operations. **(C)** Is the deployment network composed of basic modules.

Whole slide images have billions of pixels in an image, which poses computational challenges to CAD systems. Processing a WSI usually takes a significant amount of time with today's CAD systems, which is not applicable to hospital diagnosis. In order to reduce the processing time of WSIs, we first designed a chain-structured convolutional neural network to accelerate the computational speed of the network. Second, in order to improve the learning ability of the network, we introduced the idea of re-parameterization and proposed an enhanced convolutional module (ECM) by invoking RepVGG. In the network training phase, a multi-branch network structure is utilized to improve the expressiveness of the network. Finally, we introduced two channel attention mechanisms (CAM), GCT and SE, which enable the network to generate different degrees of relationships for different channels and obtain channel features with higher relevance to further improve the learning ability of the model. We named the proposed networks RepVGG-GCT and RepVGG-SE, respectively, according to the difference in attention mechanism. Figure 2 shows the training model, the re-parameterization process, and the deployment model.

As shown in Figure 2A, in the training phase, we replace each convolution with an enhanced convolution in order to compensate for the disadvantages of the chain structure. Enhanced convolution can effectively extract the feature information of different branches and then fuse the feature information of different branches to obtain the final feature information. A channel attention mechanism module is added to the output of each enhanced convolution with the purpose of making the network go automatically acquire channel features with higher relevance, thus improving the accuracy of the model. Although the multi-branch structure can obtain richer feature maps and improve the learning ability and expressiveness of the model, these structures tend to cause higher memory access costs and, thus, reduce the model running speed. To solve this problem, the multi-branch structure is merged and reduced to a common convolution using re-parameterization in the deployment phase, as shown in Figures 2B,C. In Figure 2C, the multi-branch structure is all reduced to a basic module (BM), using the idea of re-parameterization which preserves the accuracy of the multi-branch structure and exploits the computational speed of the chain structure, so that the proposed network can operate efficiently. The enhanced convolution, re-parameterization, and channel attention mechanisms are described in detail next.

1) **Enhanced Convolution Module (ECM):** Although the simple model structure can operate efficiently, the model is not expressive enough. To compensate for this, we carefully designed an enhanced convolution embedding into our network structure. In order to eliminate the influence of size on the recognition results, multiple different filter sizes are used at one time to capture multiple concepts with different ranges, so that the network can choose the required features by itself.

We first chose a 3×3 convolution to ensure the most basic performance. Second, in order to learn features with multiple receptive fields, we added a 1×1 convolution branch to increase the expressiveness of the model. Normal convolution can be denoted as:


(1) $$\begin{array}{l} F=K*X+B_{s}, \end{array}$$

where *X*, *F*, *K*, and *B*_*s*_ are the input feature maps, output feature maps, and the weights and biases of normal convolution.

Based on the success of ResNet (He et al., 2016), residual connectivity can effectively mitigate gradient disappearance, increase feature reuse, and improve the expressiveness of the model. Therefore, we also incorporated residual connectivity in our network. Note, that each convolution layer is followed by a BN layer. The BN can be expressed as:


(2) $$\begin{array}{l} B_{n}\left(X\right)=\left(X-\mu \right)\frac{\gamma }{\sigma }+\beta , \end{array}$$

Where *X*, *B*_*n*_(*X*) and {μ, σ, γ, β} as the input feature maps, output feature maps and accumulated mean, standard deviation, learned scaling factor, bias of the BN layer.

2) **Re-parameterization:** Since the targeted optimization of 3×3 convolutions in mainstream computing platforms, such as NVIDIA's GPU and Intel's CPU. In the model inference stage, all the networks are converted to 3×3 convolutions using a fusion strategy, which facilitates the deployment and acceleration of implementing the model. A re-parameterization operation is accomplished for the ECM. The first step performs the convolutional layer and the BN fusion. This operation is performed in the infer phase of many deep learning frameworks. The convolution layer and the BN fusion formula is as follows:


(3) $$\begin{array}{l} B_{n}\left(F\right)=\left(K*X+B_{s}-\mu \right)\frac{\gamma }{\sigma }+\beta \\ \;=X*K\frac{\gamma }{\sigma }+\left(\frac{\left(B_{s}-\mu \right)\gamma }{\sigma }+\beta \right) \end{array}$$

At this point, we can convert the convolution layer and BN into a vector with bias. Denote by { *K*′, $B_{s}^{\prime }$ } as the { weight, bias } of convolution after fusion.


(4) $$\begin{array}{l} K\prime =K\frac{\gamma }{\sigma }, \end{array}$$

(5) $$\begin{array}{l} B_{s}^{\prime }=\frac{\left(B_{s}-\mu \right)\gamma }{\sigma }+\beta , \end{array}$$

Therefore, it can be derived to verify that,


(6) $$\begin{array}{l} B_{n}\left(F\right)=K\prime *X+B_{s}^{\prime }, \end{array}$$

Because the whole ECM contains 1×1 convolution and identity, we convert the two branches to 3×3 convolutions. For the 1×1 convolution branch, the whole conversion process is to replace the 1×1 convolution kernel with the 3×3 convolution kernel by moving the value in the 1×1 convolution kernel to the central point of the 3×3 convolution kernel. For the identity branch, the branch does not change the value of the input feature maps. Therefore, we can set up a 3×3 convolution kernel with the central point of 1 value and the other points with 0 value. When the 1×1 convolution and identity are converted to 3×3 convolutions, the final three branches are all 3×3 convolutions.

Finally, the weights and biases of all branches are combined to obtain a fused 3×3 convolution layer through element-wise adding operations. Since the three branches are 3×3 convolutions, the fusion of convolution kernels can be achieved by element-wise adding. In the inference stage, a single normal convolution can be used to obtain the output features.

**Figure 3:**
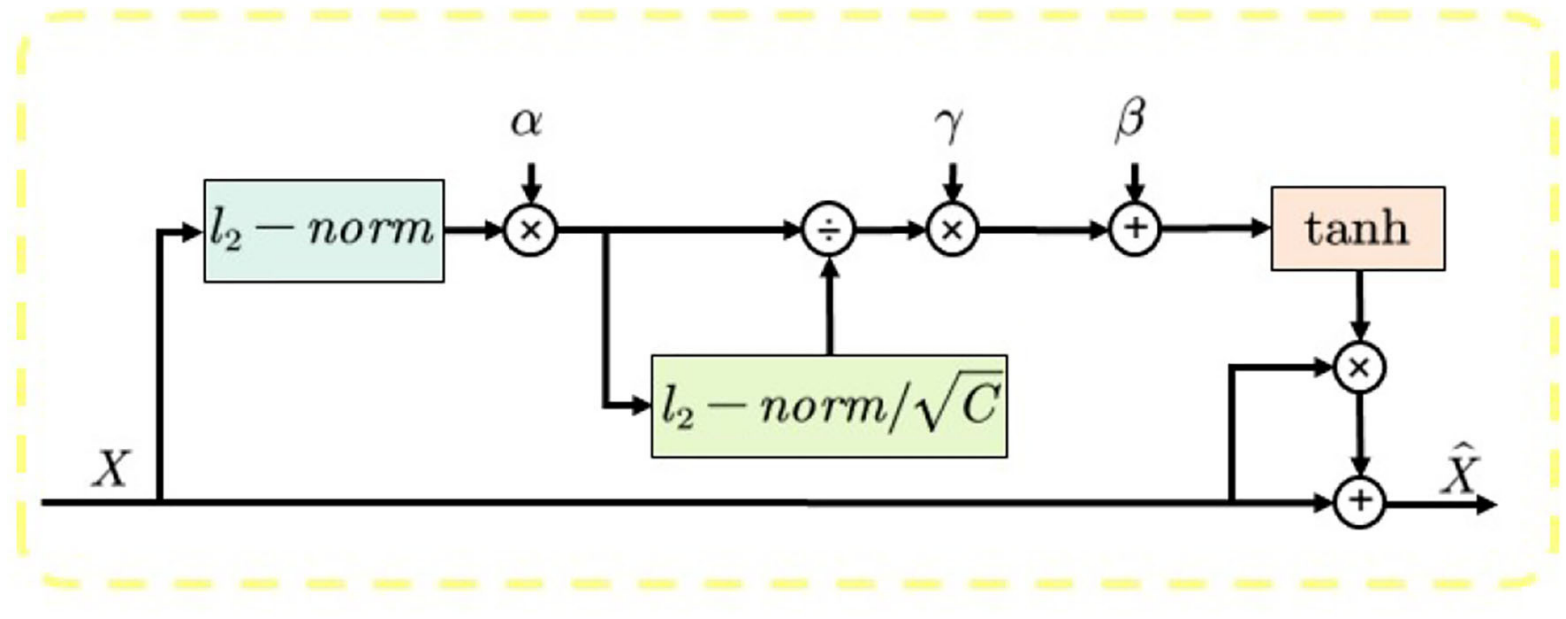
The overall structure of Gated Channel Transformation (GCT).

**Figure 4:**
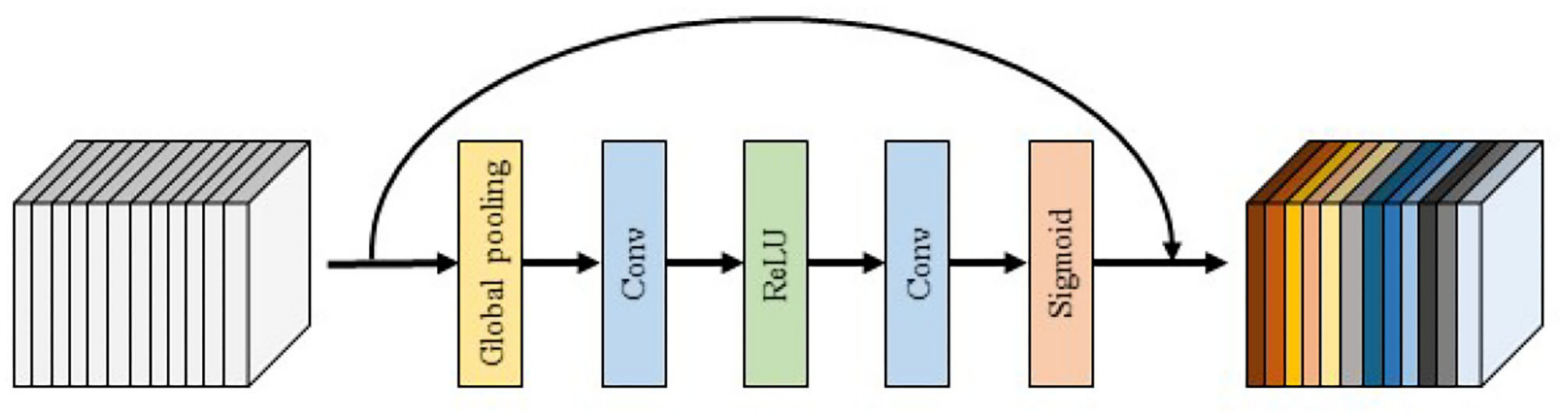
The overall structure of SE.

3) **Gated Channel Transformation (GCT):** To ensure the size and quality of the model, we chose the lightweight channel attention mechanism called GCT (Yang et al., 2020) as our attention mechanism, and the overall structure of GCT is shown in Figure 3. The module is divided into three parts. First, the global information of the input feature map is calculated using the *l*_2_ parametrization, and trainable parameters α are introduced to control the weights of each channel. During the training process, the model can automatically learn the weights of each channel. Second, the channel normalization process is performed using the *l*_2_ parametrization, which is beneficial for the training convergence. Finally, β and γ are introduced to establish the competition or cooperation between channels. When the feature weight β of a channel is activated positively, GCT will promote the features of this channel to “competition” with the features of other channels. When a channel's feature weight β is negatively activated, GCT promotes “cooperation” between the channel's features and other channels' features. When the channel features are in a “competition” relationship, the distinction between channels is large, which will motivate the model to pay more attention to the channel that it is interested in, thus improving the performance of the model.

4) **SE block:** The SE block is shown in Figure 4. In order to use the global spatial features of each channel as the representation of that channel, the global pooling is first used to generate the statistics of each channel, followed by the use of convolution and activation layers to extract the global spatial features of the channel. Finally, the Sigmoid activation function is used to map the global features of the channel to the range of 0–1 to obtain the final global spatial features. SE block improves the representation capability of the network by modeling the dependencies of each channel and adjusting features channel by channel so that the network can learn to selectively strengthen the features containing useful information and suppress useless features through global information.

### 3.2. WSI-Level HER2 Score Prediction Network

The purpose of this step is to predict HER2 scores in a WSI based on the TLCN stage. The input of the WHSPN model is a concatenated vector of the TLCN's output, which includes the predicted probabilities of image patches in a WSI. The output of the WHSPN model is the HER2 score of a WSI. Four classes including 0, 1+, 2+, 3+ were diagnosed for each WSI by pathologists, and the labels used in the WHSPN model are the whole slide HER2 scores that were derived from the clinical information.

Tile-level classification network can effectively predict the image category of each patch, and then count the percentage distribution of 0, 1+, 2+, and 3+ in the WSI tumor region, which has a certain influence on the final WSI prediction. To explore this effect, two models, Support Vector Machines (SVM) and Multilayer Perceptron (MLP), were used to explore the effect of category occupancy on the final results of WSI.

1) **Support Vector Machines (SVM):** Considering the small sample size of our training data, it is not effective to train using a large model. Due to the optimization algorithm problem of SVM, its training complexity will be highly prominent on a large dataset, while for a small dataset its model is more powerful and effective. Therefore, we used the SVM as the first scheme. The “kernel,” “gamma,” and “C” are important parameters that affect the performance of the model. The kernel function is chosen to be useful for the nonlinear hyperplane “poly.” High gamma values will try to exactly match each training dataset, which may lead to generalization errors and cause overfitting problems. Due to our small sample size, gamma = 10 was chosen. The penalty parameter C for the error term was defined as 2, which also controls the smoothing of the decision boundary and the correct balance between the classification training points.

2) **Multilayer Perceptron (MLP):** Since the data output from TLCN is linear indistinguishable data, we need to develop HER2 scoring criteria. The traditional way of defining the scoring criteria directly sets thresholds, which leads to subjective errors. MLP is a generalization of the perceptron to overcome the weakness that the perceptron cannot recognize linear indistinguishable data. Our second scheme is designed with four fully connected layers. Each layer is followed by ReLU to make it converge better.

## 4. Experiments

### 4.1. Datasets

**Figure 5:**
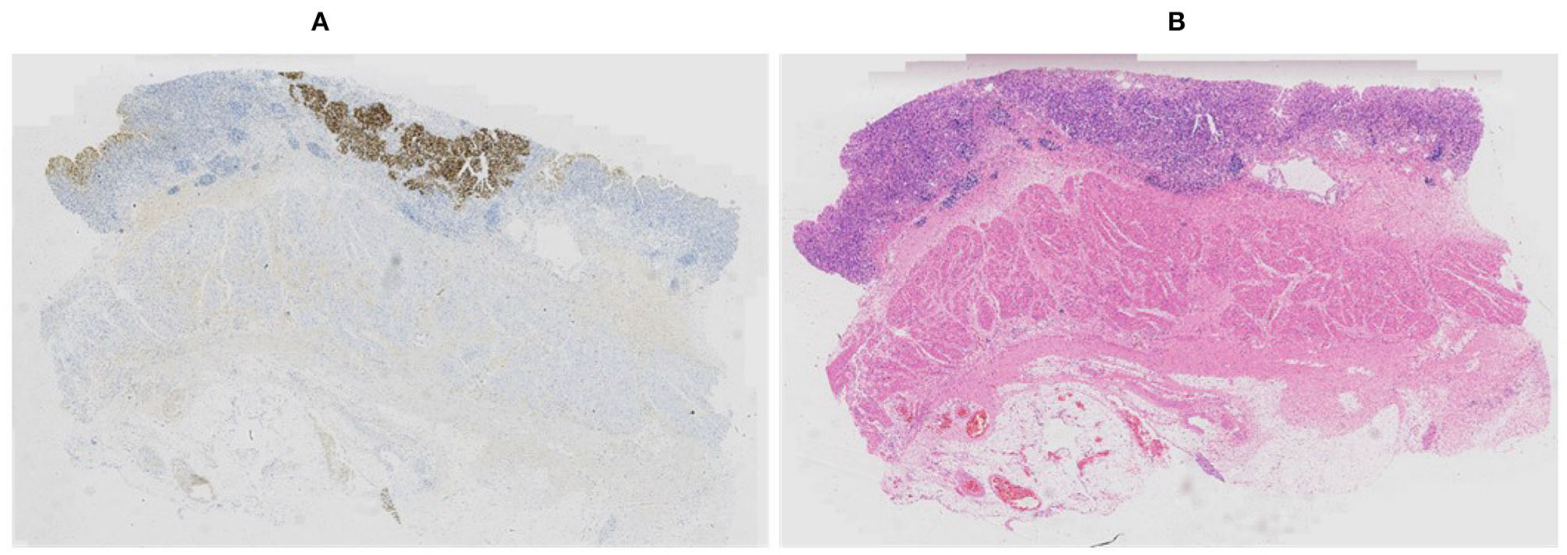
Example of corresponding sections in **(A)** Human epidermal growth factor receptor 2 (HER2) and **(B)** hematoxylin and eosin (H&E) stains.

Our proposed method was evaluated on the HER2 image dataset of Fujian Cancer Hospital. The dataset involves WSIs, including H&E and HER2 stained slides. The examples of H&E and HER2 stained slides are shown in Figure 5. H&E and HER2 stained slides are used in routine diagnostic practice for gastric cancer. The pathologist first uses H&E stained images to identify the tumor area and then determines the HER2 scores by HER2 stained slides. Our method uses only HER2 stained slides without H&E stained slides to obtain HER2 scores. A total of 183 HER2 stained WSIs from Fujian Cancer Hospital were collected, of which 75 WSIs for WHSPN training, 8 WSIs for WHSPN validation, and the remaining 100 WSIs for WHSPN testing. Ground truth (GT) is the clinical diagnosis of HER2 interpretation based on WSIs from the pathologists of Fujian Cancer Hospital. We randomly extracted 20 WSIs from the training dataset of WHSPN as the TLCN dataset and sliced these 20 WSIs into 256*256 pixels patches. In this way, we collected 17,444 patches of 0, 1+, 2+, and 3+ for training. The GT of each patch used in the TLCN model was labeled by pathologists according to its staining pattern and degree. For example, if the patch was sampled from the 1+ region, the “1+” label was given. Similarly, if the patch was sampled from the 3+ region, it will be given the label “3+.” Note that a patch's label is not necessarily equivalent to a WSI's label, because a WSI may contain patches with different labels at the same time. All patches extracted from the 20 WSIs were mixed together and the patches were divided into a training dataset, validation dataset, and test dataset in the ratio of 8:1:1. Table 2 shows the data distribution of TLCN. Table 3 shows the data distribution of WHSPN. These HER2 stained slides were obtained by UNIC PRECICE 600 scanning instrument at 40× optical magnification (0.12 μm/pixel). HER2 scoring criteria were based on Li et al. (2016), and the tumor tissues of selected cases were IHC stained for HER2 protein expression status assessment. Table 1 shows the assessment criteria.

**Table 2:** Demographics of the dataset used for Tile-level classification network (TLCN).

**TLCN Data Set**	Number of Patches(256*256pixel)		
**HER2 Score (Ground Truth)**	**Train**	**Val**	**Test**
0	5,446	673	615
1+	4,216	520	470
2+	3,902	482	437
3+	3,880	479	326
Total	17,444	2,154	1,848

**Table 3:** Demographics of the dataset used for WSI-level HER2 score prediction network (WHSPN).

**WHSPN Data Set**	**Number of WSIs**		
**HER2 Score(Ground Truth)**	**Train**	**Val**	**Test**
0	22	2	25
1+	17	2	25
2+	21	2	28
3+	15	2	22
Total	75	8	100

**Figure 6:**
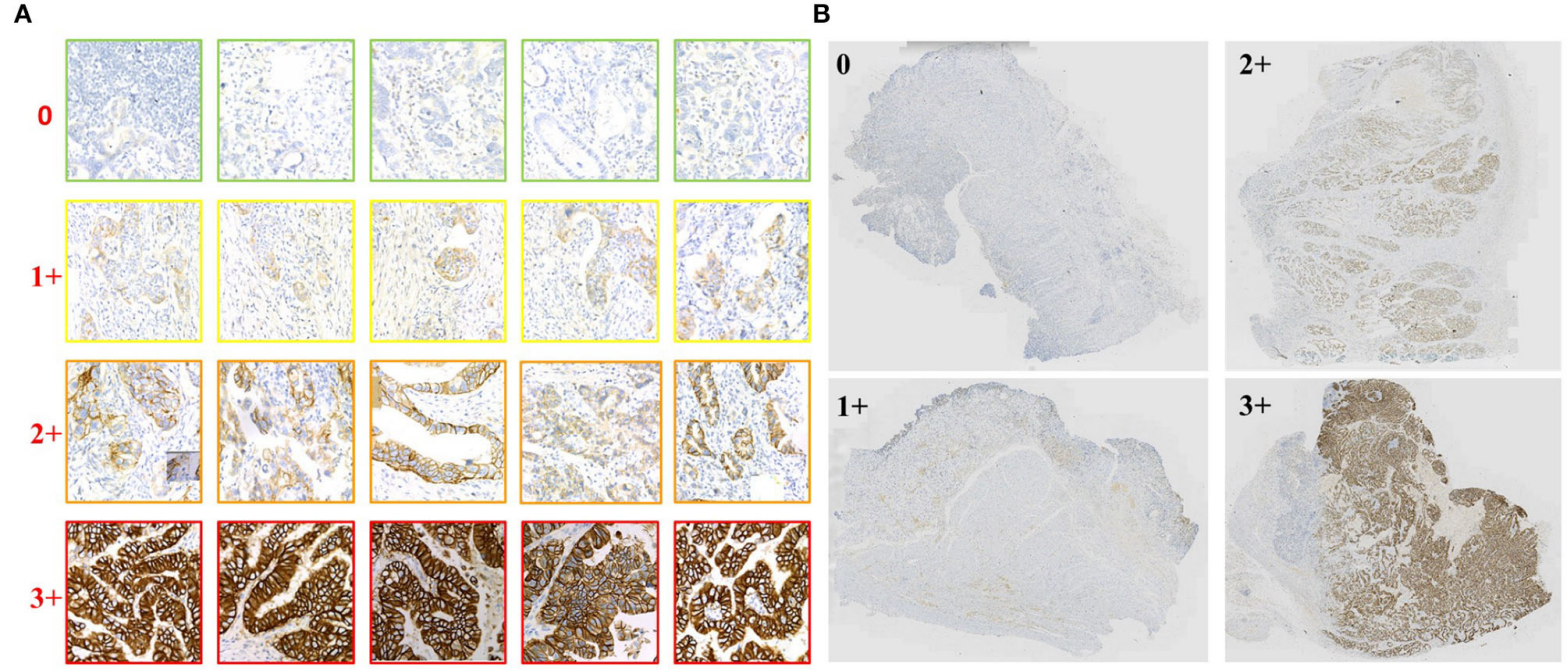
Examples of data of Tile-level classification network (TLCN) and WSI-level HER2 score prediction network (WHSPN). **(A)** Samples of the TLCN dataset, which is composed of 256*256 pixels patches cropped from Whole slide images (WSIs). **(B)** Samples of the WHSPN dataset, which is consisted of WSI with billions of pixels.

Our experiments include two parts of data. The first part is the training and validation data of TLCN. Figure 6A shows samples of the TLCN dataset, which is composed of 256*256 pixels patches cropped from WSI. The second part is the training and validation data of WHSPN. Figure 6B shows samples of the WHSPN dataset, which is consisted of WSIs with billions of pixels. Table 2 shows the data distribution of TLCN. Table 3 shows the data distribution of WHSPN.

### 4.2. Implementation Details

All models in this experiment were trained on an Ubuntu system with Nvidia RTX 2080TI GPUs. The experiments were performed with SGD as the optimizer, CrossEntropyLoss as the loss, initial learning rate set to 0.1, weight decay set to 0.001, and batch size set to 256. Two hundred epochs were performed for all experiments.

### 4.3. Evaluation Metrics

Although Accuracy is the most common and basic evaluation metric, its reference value is not high in the case of data imbalance. Therefore, in addition to the most commonly used accuracy, we also describe the performance of classifiers by precision, recall, specificity, and F1-score. Evaluation metrics are calculated as follows:


(7) $$\begin{array}{l} Accuracy=\frac{TP\;+\;TN}{TP\;+\;TN\;+\;FP\;+\;FN}. \end{array}$$

(8) $$\begin{array}{l} Precision=\frac{TP}{TP\;+\;FP}, \end{array}$$

(9) $$\begin{array}{l} Recall=\frac{TP}{TP\;+\;FN}, \end{array}$$

(10) $$\begin{array}{l} specificity=\frac{TN}{FP\;+\;TN}, \end{array}$$

(11) $$\begin{array}{l} F1-score=\frac{2}{\frac{1}{Precision}\;+\;\frac{1}{Recall}}, \end{array}$$

where TP, FP, TN, and FN denote true positive, false positive, true negative, and false negative.

### 4.4. TLCN Results

First, we compared our methods with many state-of-the-art (SOTA) methods. Table 4 shows the experimental comparison results of TLCN, in terms of experimental evaluation metrics and model parameters. We used Monte-Carlo Cross-Validation experiments to get more convincing results than a single validation. Each time, the data set is randomly divided into training and validation sets. In this way, multiple models training were carried out. Finally, the average performance value with a standard deviation of these results was used as the final results of this model. Obviously, the two proposed networks, RepVGG-GCT and RepVGG-SE, have more accurate performance in HER2 patch-level classification and can achieve higher accuracy with fewer parameters. The two networks we proposed are improved on VGGNet (Simonyan and Zisserman, 2014). The *Params* has dropped from 138.358M to about 7M, and the *Flops* has dropped from the original more than 20 G to 1.78 G. In addition, the *Accuracy*, *Precision*, *Recall*, *Specificity*, *F*1 − *score*, and speed has been improved with fewer parameters. Note that the *time* in the table means the time required for the network to infer a patch.

**Table 4:** Classification performance of our RepVGG-SE model and RepVGG-GCT model and several recently published classification approaches on the TLCN datasets.

**Methods**	**Accuracy**	**Precision**	**Recall**	**Specificity**	**F1**	**Params/M**	**Flops/G**	**Time/ms**
VGGNet (Simonyan and Zisserman, 2014)	92.89 ± 0.35	90.44 ± 0.48	90.26 ± 0.51	98.25 ± 0.09	90.35 ± 0.48	138.358	20.21	7.853
Inception-v3 (Szegedy et al., 2016)	92.33 ± 0.54	89.95 ± 0.63	89.50 ± 0.87	98.09 ± 0.16	89.73 ± 0.71	23.835	3.86	13.64
ResNet-50 (He et al., 2016)	92.59 ± 0.28	90.66 ± 0.25	89.37 ± 0.61	98.08 ± 0.07	90.01 ± 0.39	25.557	5.38	7.432
DenseNet-169 (Huang et al., 2017)	92.88 ± 0.16	90.58 ± 0.34	89.96 ± 0.34	98.25 ± 0.05	90.27 ± 0.18	14.149	4.46	22.63
ShufleNet-v2 (Ma et al., 2018)	92.39 ± 0.14	90.17 ± 0.19	89.33 ± 0.41	98.07 ± 0.07	89.75 ± 0.22	**3.504**	0.396	6.653
MobileNetV3 (Howard et al., 2019)	92.10 ± 0.32	89.79 ± 0.58	89.11 ± 1.02	98.05 ± 0.16	89.44 ± 0.49	5.483	**0.296**	6.557
EfficientNet-B3 (Tan and Le, 2019)	92.87 ± 0.28	90.77 ± 0.22	89.86 ± 0.53	98.20 ± 0.10	90.31 ± 0.37	12.233	1.29	13.87
RepVGG-SE(ours)	93.03 ± 0.35	90.91 ± 0.58	90.30 ± 0.51	98.26 ± 0.10	90.60 ± 0.47	7.316	1.78	5.283
RepVGG-GCT(ours)	**93.16 ± 0.10**	**90.92 ± 0.19**	**90.48 ± 0.34**	**98.31 ± 0.07**	**90.70 ± 0.19**	7.049	1.78	**5.185**

*The bold values mean the Best results*.

Comparing our two networks with some more advanced lightweight networks such as Mobilenent (Howard et al., 2019) and ShuffleNet (Ma et al., 2018), the model parameters of our proposed networks are only a little larger than these two networks, but the accuracy has been effectively improved. Furthermore, the speed is faster than these two lightweight networks. In MobileNetV3, the author continues to use the depthwise separable convolution. In theory, the depthwise separable convolution requires less computation. However, because the operation intensity of depthwise separable convolution (the ratio of Flops to memory access) is too low, it is difficult to make effective use of hardware. As a result, MobileNetV3 has a smaller number of parameters but is slower compared to our models. Additionally, in the layer structure of depthwise separable convolution, each depthconv has no cross-channel information, and even if it is compensated by pointconv afterward, pointconv again lacks spatial association information, which results in a more accurate TLCN model than MobileNetV3.

We further analyzed the performance of our methods in each category separately. From Table 5, we can find that the *Precision*, *Recall*, *Specificity*, and *F*1 − *scores* of 2+ and 3+ have achieved good results, and the categories of 0 and 1+ are easy to be confused. However, in the daily diagnosis of doctors, 0 and 1+ are determined to be negative. Therefore, we combined 0 and 1+ into one category, which is negative. It is determined that 2+ is equivocal and 3+ is positive. According to the experimental results, all indicators have been significantly improved compared with the previous studies.

**Table 5:** Classification performance of our RepVGG-GCT model and RepVGG-SE model in four classes and three classes.

		**FOUR CLASSES**				**THREE CLASSES**		
**Methods**		**0**	**1+**	**2+**	**3+**	**0/1+ (Negative)**	**2+ (Equivocal)**	**3+ (Positive)**
RepVGG-GCT	Precision	85.2	82	90.2	99.5	93.1	90.3	99.6
	Recall	84.6	71.9	90.8	99.5	91.5	94.4	99.2
	Specificity	98	98.4	99.1	1	98.1	99	1
	F1	84.9	76.6	90.5	99.5	92.3	92.3	99.4
RepVGG-SE	Precision	83.7	82.6	90.4	98.9	92.7	92.1	99.2
	Recall	88.8	77.7	90.4	99.5	90.3	92.5	99.4
	Specificity	97.7	98.4	99.1	99.9	98.1	99.3	99.9
	F1	86.2	80.1	90.4	99.2	91.5	92.3	99.3

### 4.5. WHSPN Results

We combined the two methods proposed on TLCN with the two methods proposed on WHSPN. WHSPN creates a vector according to the proportion of 0, 1+, 2+, 3+ categories output by TLCN, so as to get the final HER2 score of WSI. Since the HER2 score only evaluates the tumor cells, noise and normal are not input to the second network. Similar to the TLCN experiment, the experiments were performed by splitting and combining 0 and 1+, respectively, and the experimental accuracy is shown in Table 6.

**Table 6:** Final HER2 evaluation results for the combination of TLCN and WHSPN.

**Accuracy on WSI-level of WHSPN-HER2 scoring (in %)**				
**Classification**	**FOUR CLASSES**		**THREE CLASSES**	
**Methods**	**MLP**	**SVM**	**MLP**	**SVM**
RepVGG-GCT	**86**	84	**94**	92
RepVGG-SE	83	83	90	91

*The bold values mean the Best results*.

**Figure 7:**
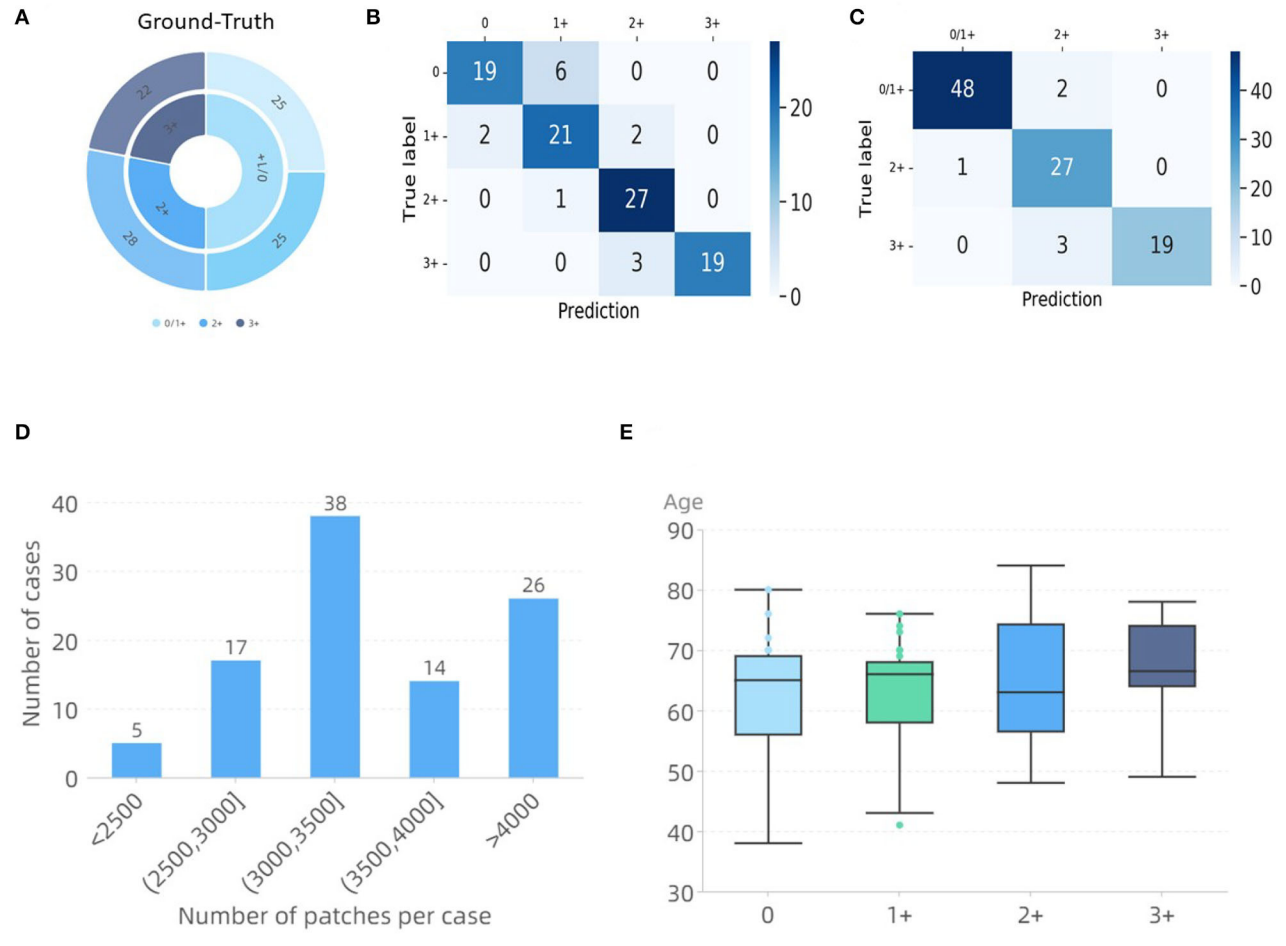
WHSPN dataset validation results. **(A)** Ground-Truth. **(B)** Confusion matrix diagram of four classes. **(C)** Confusion matrix diagram of three classes. **(D)** Distribution of the number of tiles per case. **(E)** Ages distribution of the cases used in this experiment.

When comparing the developed methods, the method of RepVGG-GCT+MLP was found to obtain the best accuracy. Since the patches of the final test WSIs do not appear in the training set, its significantly high accuracy indicates that the modified model has a good learning ability for HER2 scores. In addition, we hope to use the existing data as much as possible to train a network with better generalization performance with a small amount of data, because of the scarcity of gastric cancer HER2 data. We only used 83 WSIs in total to train the TLCN and WHSPN networks, and finally verified the feasibility of 100 WSIs. Figure 7 shows the final validation results. It shows a confusion matrix of four classes and three classes using the RepVGG-GCT+MLP method. Through the confusion matrix, it can be seen that the categories of 0 and 1+ are more likely to be confused, but this does not affect the doctor's daily diagnosis since both of them are negative. Figure 7D lists the number of image patches of 100 WSIs used for verification in this experiment, which reflects the variety in WSI sizes. Figure 7E shows the age distribution of these 100 patients. The results demonstrate the feasibility of our proposed network in the prediction of HER2 scores.

### 4.6. Ablation Study

To verify the effectiveness of the proposed module and the feasibility of model re-parameterization, we performed ablation experiments. Table 7 shows the effect of ECM, GCT, and re-parameterization. Similarly, we used Monte-Carlo Cross-Validation in our ablation experiments to evaluate the performance of each module.

**Table 7:** Results of selecting block.

**Block**	**ECM**	**GCT**	**Re-parameterization**	**Accuracy**	**Precision**	**Recall**	**Specificity**	**F1**	**Params/M**	**Flops/G**	**Time**
Baseline				56.11 ± 0	9.35 ± 0	16.67 ± 0	83.33 ± 0	11.98 ± 0	**7.031**	**1.78**	**1.479**
		✓		56.11 ± 0	9.35 ± 0	16.67 ± 0	83.33 ± 0	11.98 ± 0	7.045	**1.78**	5.232
	✓			92.61 ± 0.17	90.38 ± 0.50	89.67 ± 0.25	98.16 ± 0.06	90.02 ± 0.24	7.836	1.99	6.449
	✓		✓	92.61 ± 0.17	90.38 ± 0.50	89.67 ± 0.25	98.16 ± 0.06	90.02 ± 0.24	7.036	**1.78**	1.691
	✓	✓		**93.16 ± 0.10**	**90.92 ± 0.19**	**90.48 ± 0.34**	**98.31 ± 0.07**	**90.70 ± 0.19**	7.849	1.99	10.243
	✓	✓	✓	**93.16 ± 0.10**	**90.92 ± 0.19**	**90.48 ± 0.34**	**98.31 ± 0.07**	**90.70 ± 0.19**	7.049	**1.78**	5.185

*The bold values mean the Best results*.

#### 4.6.1. Effect of the Enhanced Convolution Module

When ECM is not added, we can see that the speed of the model is very fast and the amount of parameters is small. However, it hardly has the ability to learn with simple structure. After adding ECM, the *Accuracy* of the model is improved from 56.11% to 92.61% by 36.5%. *Precision* is improved from 9.35% to 90.38%% by 81.03%. The *recall* is improved from 16.67% to 89.67% by 73%. *Specificity* is improved from 83.33% to 98.16% by 14.83%, and the *F*1 − *score* is improved from 11.98% to 90.02% by 78.04%.

#### 4.6.2. Effect of the Channel Attention Module

After adding GCT, the *Accuracy* of the model is improved from 92.61% to 93.16% by 0.55%. *Precision* is improved from 90.38% to 90.92% by 0.54%. The *recall* is improved from 89.67% to 90.48% by 0.81%. *Specificity* is improved from 98.16% to 98.31% by 0.15%, and *F*1 − *score* is improved from 90.02% to 90.70% by 0.68%.

#### 4.6.3. Effect of the Re-parameterization

Since the basic module cannot be re-parameterized, the ablation experiment of re-parameterized was just performed using ECM. In order to verify the effectiveness of the deployed networks after re-parameterization, we compared the network with and without re-parameterization. It can be seen that the parameters of the re-parameterized model decreased, and the speed is improved without any loss of precision. Compared with the “baseline+ECM” without re-parameterization, the model after re-parameterization has no loss in *Accuracy*, *Precision*, *Recall*, *Specificity*, and *F*1 − *score*. At the same time, *Params* has dropped from 7.836 to 7.036 M. *Flops* has dropped from 1.99 to 1.78 G, and *Time* has dropped from 6.449 to 1.691 ms. Compared with the “baseline+ECM+GCT” without re-parameterization, the model after re-parameterization has no loss in *Accuracy*, *Precision*, *Recall*, *Specificity*, and *F*1 − *score*. At the same time, *Params* has dropped from 7.849 to 7.049 M. *Flops* has dropped from 1.99 to 1.78 G, and *Time* has dropped from 10.243 to 5.185 ms.

## 5. Discussion

The current interpretation of HER2 is only semi-quantitative by pathologists and requires artificial intelligence for quantitative HER2 analysis in particular. We proposed a novel automatic HER2 scoring framework in this study. It can not only give the prediction results of HER2 score at the WSIs level but also obtain specific quantitative results to assist doctors in interpretation. Avoiding the tedious process of comparing H&E stained slides and HER2 stained slides and the subjective difference of semi-quantitative scores, an automatic method is of far-reaching significance in assisting the interpretation of HER2 in gastric cancer. The proposed method can achieve high accuracy by using a network with fewer parameters, and it can diagnose in real time. Comparing the previously advanced networks on the same dataset, our proposed network has better HER2 classification results. In addition, the advantages of our model in parameters and inference time show its superiority in HER2 interpretation. For instance, pruning and quantization can also allow reducing memory footprint or increasing speed. Pruning reduces the number of parameters by removing redundant, unimportant connections that are not sensitive to performance. The disadvantage is that pruning is slightly effective compared to directly modifying the model structure. Quantification can significantly narrow the size of a deep neural network by reducing the number of bits used. The disadvantage is that quantized weights make it harder for the neural network to converge, which may result in a loss of accuracy. The idea of re-parameterization can be understood as re-modifying the structure of the model to reduce the size of the whole model, thus saving computation time. Additionally, this change does not bring any decrease in accuracy.

Another strength of our strategy is that in order to avoid segmentation and manual intervention, only automatic classification algorithms are used, reducing the complexity of the analysis. The current mainstream approach for HER2 interpretation is to perform feature extraction, cell membrane, and nucleus segmentation or detection, followed by classification. Due to its complex process, it usually relies on extensive and accurate annotations (Vandenberghe et al., 2017; Khameneh et al., 2019) or manual intervention (Masmoudi et al., 2009; Brügmann et al., 2012). However, manually labeling of cell membrane and nucleus is time-consuming and challenging work. For example, a WSI usually has thousands of nuclei and cell membranes. In addition, in some studies, HER2 interpretation was simply implemented on the region-of-interest (ROI) or patch and did not realize the HER2 interpretation on a WSI (Saha and Chakraborty, 2018; Singh and Mukundan, 2018). We try to optimize this workflow using the proposed classification method to reduce the annotation difficulty for pathologists, which also largely reduces the complexity of the analysis. The results demonstrated that our strategy can work well on this task. Table 8 summarizes the comparison of other state-of-the-art methods with ours. Our annotation is relatively simple and finally achieved HER2 interpretation on WSIs. Note that the accuracy of the Remarks part in Table 8 corresponds to its final interpretation result. For example, if the HER2 score calculation on WSIs is not implemented, the result represents the classification accuracy of patches.

**Table 8:** Summary of HER2 scoring methods.

**Cancer type**	**Comparison**	**Methodology used**	**Nuclei/membrane Annotation**	**Classfication Annotation**	**HER2 score calculation on WSIs**	**Image type**	**Dataset**	**Patch size**	**Remarks**
Breast cancer	(Masmoudi et al., 2009)	Conventinal techniques	Yes	Yes	No	IHC	77 WSIs	ROI	81%-83% agreement
	(Brügmann et al., 2012)		Yes	Yes	No		253 WSIs	ROI	92.3% agreement
	(Cordeiro et al., 2018)		No	Yes	Yes		86 WSIs	250x250	90% accuracy
	(Vandenberghe et al., 2017)	Deep learining	Yes	Yes	Yes		74 WSIs	44x44	83% concordance
	(Singh and Mukundan, 2018)		Yes	Yes	No		52 WSIs	512x512	91.1% accuracy
	(Saha and Chakraborty, 2018)		Yes	Yes	No		79 WSIs	251x251	98.33% accuracy
	(Qaiser and Rajpoot, 2019)		Yes	Yes	Yes		86 WSIs	2048x2048	79.4% accuracy
	(Khameneh et al., 2019)		Yes	Yes	Yes		127 WSIs	512x512	87% accuracy
	(Zhang et al., 2020)		Yes	Yes	Yes		285 WSIs	2048x2048	95% accuracy
Gastric cancer	(Sharma et al., 2017)		No	Yes	No	H&E	11 WSIs	512x512	69.90 accuracy
	ours		No	Yes	Yes	IHC	183 WSIs	256x256	94% accuracy

Our study also has some limitations. The purpose of this study is to obtain HER2 intelligent quantitative score only by using HER2 stained slides without H&E stained slides. However, HER2 stained slides are expensive and the production process is complex. Future study will continue to explore whether HER2 score can be interpreted from H&E stained slides only, without HER2 stained slides. Another limitation is that the images we used were all from the same scanner and do not provide good evidence of the generalizability of our models. Although there are differences in the results of stained slides from one lab to another, these differences are slowly diminishing due to enhanced technology. We hope that the proposed method can be evaluated using images from different hospitals in the future. However, due to the paucity of images, we have not yet done the validation. In addition, we analyzed our results and found that our method interprets higher on slices with better imaging than slices with poor imaging (e.g., folding, squeezing).

Due to the particularity of the medical field, the success of the artificial intelligence model largely depends on the trust of the pathologist in the results of AI. However, if pathologists rely too heavily on software, it may change their initial diagnostic intent. Sometimes pathologists will trust their own interpretations more than the data derived from the AI. In addition to improving the accuracy of the models, visualization (e.g., Quantitative indicators are given by outputting a matrix of the proportion of each category in tumor cells) should be added to increase the trust of pathologists in AI. Despite these limitations, this study appears to the potential of deep learning in HER2 interpretation as an automated screening tool to provide a clinical aid to physicians.

## Data Availability Statement

The original contributions presented in the study are included in the article/supplementary files, further inquiries can be directed to the corresponding author.

## Author Contributions

ZHa, JL, TW, ZHu, YH, YD, QG, MD, TT, and GC: concept and design. ZHa, JL, HZ, JW, MC, and TT: acquisition of data. ZHa, JL, TW, ZHu, QG, and TT: model design. ZHa, JL, TW, ZHu, YH, YD, and TT: data analysis. ZHa, JL, TW, ZHu, YH, YD, TT, and GC: manuscript drafting. ZHu, JL, TW, ZHu, YH, YD, HZ, JW, MC, HJ, R-GL, QG, MD, TT, and GC: approval. All authors contributed to the article and approved the submitted version.

## Funding

This study was supported in part by the National Natural Science Foundation of China under Grant 61901120 and Grant 62171133, the Science and Technology Program of Fujian Province of China under Grant 2019YZ016006, and Health and Family Planning Research Talent Training Program of Fujian Province under Grant 2020GGB009.

## Conflict of Interest

QG and TT were employed by the company Imperial Vision Technology. The remaining authors declare that the research was conducted in the absence of any commercial or financial relationships that could be construed as a potential conflict of interest.

## Publisher's Note

All claims expressed in this article are solely those of the authors and do not necessarily represent those of their affiliated organizations, or those of the publisher, the editors and the reviewers. Any product that may be evaluated in this article, or claim that may be made by its manufacturer, is not guaranteed or endorsed by the publisher.

## References

[B1] Ai S. Li C. Li X. Jiang T. Grzegorzek M. Sun C.. (2021). A state-of-the-art review for gastric histopathology image analysis approaches and future development. Biomed. Res Int. 2021, 6671417. 10.1155/2021/6671417 34258279 PMC8257332

[B2] Alatab S. Sepanlou S. Ikuta K. S. Vahedi H. Naghavi M. (2020). The global, regional, and national burden of inflammatory bowel disease in 195 countries and territories, 1990–2017: a systematic analysis for the global burden of disease study 2017. Lancet Gastroenterol. Hepatol. 5, 17–30. 10.1016/S2468-1253(19)30333-4 31648971 PMC7026709

[B3] Andre E. Brett K. Novoa R. A. Justin K. Swetter S. M. Blau H. M.. (2019). Dermatologist-level classification of skin cancer with deep neural networks. Nature 542, 115–118. 10.1038/nature21056 28117445 PMC8382232

[B4] Bang Y.-J. Van Cutsem E. Feyereislova A. Chung H. C. Shen L. Sawaki A.. (2010). Trastuzumab in combination with chemotherapy versus chemotherapy alone for treatment of her2-positive advanced gastric or gastro-oesophageal junction cancer (toga): a phase 3, open-label, randomised controlled trial. Lancet 376, 687–697. 10.1016/S0140-6736(10)61121-X 20728210

[B5] Brügmann A. Eld M. Lelkaitis G. Nielsen S. Grunkin M. Hansen J. D.. (2012). Digital image analysis of membrane connectivity is a robust measure of her2 immunostains. Breast Cancer Res. Treat. 132, 41–49. 10.1007/s10549-011-1514-2 21512768

[B6] Contest (2016). Her2 Scoring Contest. Available online at: https://warwick.ac.uk/fac/cross_fac/tia/data/her2contest/.

[B7] Cordeiro C. Q. Ioshii S. O. Alves J. H. Oliveira L. F. (2018). An automatic patch-based approach for her-2 scoring in immunohistochemical breast cancer images using color features. arXiv preprint arXiv:1805.05392. 10.5753/sbcas.2018.3685

[B8] Coudray N. Ocampo P. S. Sakellaropoulos T. Narula N. Snuderl M. Fenyö D.. (2018). Classification and mutation prediction from non-small cell lung cancer histopathology images using deep learning. Nat. Med. 24, 1559–1567. 10.1038/s41591-018-0177-5 30224757 PMC9847512

[B9] Ding X. Guo Y. Ding G. Han J. (2019). Acnet: strengthening the kernel skeletons for powerful cnn via asymmetric convolution blocks, in Proceedings of the IEEE/CVF International Conference on Computer Vision (Seoul: IEEE), 1911–1920.

[B10] Ding X. Zhang X. Han J. Ding G. (2021a). Diverse branch block: building a convolution as an inception-like unit, in Proceedings of the IEEE/CVF Conference on Computer Vision and Pattern Recognition (Nashville, TN: IEEE), 10886–10895.

[B11] Ding X. Zhang X. Ma N. Han J. Ding G. Sun J. (2021b). Repvgg: making vgg-style convnets great again, in Proceedings of the IEEE/CVF Conference on Computer Vision and Pattern Recognition, 13733–13742.

[B12] Ellis C. Dyson M. Stephenson T. Maltby E. (2005). Her2 amplification status in breast cancer: a comparison between immunohistochemical staining and fluorescence *in situ* hybridisation using manual and automated quantitative image analysis scoring techniques. J. Clin. Pathol. 58, 710–714. 10.1136/jcp.2004.023424 15976337 PMC1770709

[B13] Garcia E. Hermoza R. Castanon C. B. Cano L. Castillo M. Castanneda C. (2017). Automatic lymphocyte detection on gastric cancer ihc images using deep learning, in 2017 IEEE 30th International Symposium on Computer-Based Medical Systems (CBMS) (Thessaloniki: IEEE), 200–204.

[B14] He K. Zhang X. Ren S. Sun J. (2016). Deep residual learning for image recognition, in Proceedings of the IEEE Conference on Computer Vision and Pattern Recognition (Las Vegas, NV: IEEE), 770–778.

[B15] Hinton G. Vinyals O. Dean J. (2015). Distilling the knowledge in a neural network. arXiv preprint arXiv:1503.02531. 10.48550/arXiv.1503.02531

[B16] Howard A. Sandler M. Chu G. Chen L.-C. Chen B. Tan M.. (2019). Searching for mobilenetv3, in Proceedings of the IEEE/CVF International Conference on Computer Vision (Seoul: IEEE), 1314–1324.

[B17] Huang G. Liu Z. Van Der Maaten L. Weinberger K. Q. (2017). Densely connected convolutional networks, in Proceedings of the IEEE Conference on Computer Vision and Pattern Recognition (Honolulu, HI: IEEE), 4700–4708.

[B18] Iizuka O. Kanavati F. Kato K. Rambeau M. Arihiro K. Tsuneki M. (2020). Deep learning models for histopathological classification of gastric and colonic epithelial tumours. Sci. Rep. 10, 1–11. 10.1038/s41598-020-58467-9 32001752 PMC6992793

[B19] Khameneh F. D. Razavi S. Kamasak M. (2019). Automated segmentation of cell membranes to evaluate her2 status in whole slide images using a modified deep learning network. Comput. Biol. Med. 110, 164–174. 10.1016/j.compbiomed.2019.05.020 31163391

[B20] Korkmaz S. A. Binol H. (2018). Classification of molecular structure images by using ann, rf, lbp, hog, and size reduction methods for early stomach cancer detection. J. Mol. Struct. 1156, 255–263. 10.1016/j.molstruc.2017.11.093

[B21] Kosaraju S. C. Hao J. Koh H. M. Kang M. (2020). Deep-hipo: multi-scale receptive field deep learning for histopathological image analysis. Methods 179, 3–13. 10.1016/j.ymeth.2020.05.012 32442672

[B22] Li J. Ren G. Teng X. (2016). Guidelines for the detection of gastric cancer her2 (2016 edition). Chin. J. Pathol45, 528–532. 10.3760/cma.j.issn.0529-5807.2016.08.007 27510777

[B23] Li Y. Li X. Xie X. Shen L. (2018). Deep learning based gastric cancer identification, in 2018 IEEE 15th International Symposium on Biomedical Imaging (ISBI 2018) (Washington, DC: IEEE), 182–185.

[B24] Liu B. Yao K. Huang M. Zhang J. Li Y. Li R. (2018a). Gastric pathology image recognition based on deep residual networks, in 2018 IEEE 42nd Annual Computer Software and Applications Conference (COMPSAC), Volume 2 (Tokyo: IEEE), 408–412.

[B25] Liu B. Zhang M. Guo T. Cheng Y. (2018b). Classification of gastric slices based on deep learning and sparse representation, in 2018 Chinese Control And Decision Conference (CCDC) (Shenyang: IEEE), 1825–1829.

[B26] Ma N. Zhang X. Zheng H.-T. Sun J. (2018). Shuffenelt v2: practical guidelines for efficient cnn architecture design, in Proceedings of the European Conference on Computer Vision (ECCV). (Munich), 116–131.

[B27] Masmoudi H. Hewitt S. M. Petrick N. Myers K. J. Gavrielides M. A. (2009). Automated quantitative assessment of her-2/neu immunohistochemical expression in breast cancer. IEEE Trans. Med. Imaging 28, 916–925. 10.1109/TMI.2009.2012901 19164073 PMC7238291

[B28] Nan Y. Coppola G. Liang Q. Zou K. Sun W. Zhang D.. (2017). Partial labeled gastric tumor segmentation via patch-based reiterative learning. arXiv preprint arXiv:1712.07488. 10.48550/arXiv.1712.07488

[B29] Qaiser T. Rajpoot N. M. (2019). Learning where to see: a novel attention model for automated immunohistochemical scoring. IEEE Trans. Med. Imaging 38, 2620–2631. 10.1109/TMI.2019.2907049 30908205

[B30] Qiu L. (2016). Chinese expert consensus on molecularly targeted therapy for her2-positive advanced gastric cancer (2016 edition). Chin. Clin. Oncol. 21, 831–839.

[B31] Saha M. Chakraborty C. (2018). Her2net: a deep framework for semantic segmentation and classification of cell membranes and nuclei in breast cancer evaluation. IEEE Trans. Image Process. 27, 2189–2200. 10.1109/TIP.2018.2795742 29432100

[B32] Schmell S. Zakrzewski F. de Back W. Weigert M. Schmidt U. Wenke T.. (2020). An interpretable automated detection system for fish-based her2 oncogene amplification testing in histo-pathological routine images of breast and gastric cancer diagnostics. arXiv preprint arXiv:2005.12066. 10.48550/arXiv.2005.12066

[B33] Sharma H. Zerbe N. Klempert I. Hellwich O. Hufnagl P. (2017). Deep convolutional neural networks for automatic classification of gastric carcinoma using whole slide images in digital histopathology. Comput. Med. Imaging Graphics 61, 2–13. 10.1016/j.compmedimag.2017.06.001 28676295

[B34] Simonyan K. Zisserman A. (2014). Very deep convolutional networks for large-scale image recognition. arXiv preprint arXiv:1409.1556. 10.48550/arXiv.1409.1556

[B35] Singh P. Mukundan R. (2018). A robust her2 neural network classification algorithm using biomarker-specific feature descriptors, in 2018 IEEE 20th International Workshop on Multimedia Signal Processing (MMSP) (Vancouver, BC: IEEE), 1–5.

[B36] Song Z. Zou S. Zhou W. Huang Y. Shao L. Yuan J.. (2020). Clinically applicable histopathological diagnosis system for gastric cancer detection using deep learning. Nat. Commun. 11, 1–9. 10.1038/s41467-020-18147-8 32855423 PMC7453200

[B37] Sun C. Li C. Zhang J. Kulwa F. Li X. (2020). Hierarchical conditional random field model for multi-object segmentation in gastric histopathology images. Electron. Lett. 56, 750–753. 10.1049/el.2020.0729

[B38] Sun M. Zhang G. Dang H. Qi X. Zhou X. Chang Q. (2019). Accurate gastric cancer segmentation in digital pathology images using deformable convolution and multi-scale embedding networks. IEEE Access 7, 75530–75541. 10.1109/ACCESS.2019.2918800

[B39] Szegedy C. Vanhoucke V. Ioffe S. Shlens J. Wojna Z. (2016). Rethinking the inception architecture for computer vision, in Proceedings of the IEEE Conference on Computer Vision and Pattern Recognition (Las Vegas, NV: IEEE), 2818–2826.

[B40] Tan M. Le Q. (2019). Efficientnet: rethinking model scaling for convolutional neural networks, in International Conference on Machine Learning. (Long Beach, California: PMLR), 6105–6114.

[B41] Vandenberghe M. E. Scott M. L. Scorer P. W. Söderberg M. Balcerzak D. Barker C. (2017). Relevance of deep learning to facilitate the diagnosis of her2 status in breast cancer. Sci. Rep. 7, 1–11. 10.1038/srep45938 28378829 PMC5380996

[B42] Wang S. Zhu Y. Yu L. Chen H. Lin H. Wan X.. (2019). Rmdl: recalibrated multi-instance deep learning for whole slide gastric image classification. Med. Image Anal. 58, 101549. 10.1016/j.media.2019.101549 31499320

[B43] Yang Z. Zhu L. Wu Y. Yang Y. (2020). Gated channel transformation for visual recognition, in Proceedings of the IEEE/CVF Conference on Computer Vision and Pattern Recognition, 11794–11803.

[B44] Zakrzewski F. de Back W. Weigert M. Wenke T. Zeugner S. Mantey R.. (2019). Automated detection of the her2 gene amplification status in fluorescence *in situ* hybridization images for the diagnostics of cancer tissues. Sci. Rep. 9, 1–12. 10.1038/s41598-019-44643-z 31160649 PMC6546913

[B45] Zhang J. Tian K. Dong P. Shen H. Yan K. Yao J.. (2020). Microscope based her2 scoring system. arXiv preprint arXiv:2009.06816. 10.48550/arXiv.2009.06816

[B46] Zhang X. Zeng H. Zhang L. (2021). Edge-oriented convolution block for real-time super resolution on mobile devices, in Proceedings of the 29th ACM International Conference on Multimedia, 4034–4043.

[B47] Zhou X. Li C. Rahaman M. M. Yao Y. Ai S. Sun C.. (2020). A comprehensive review for breast histopathology image analysis using classical and deep neural networks. IEEE Access 8, 90931–90956. 10.1109/ACCESS.2020.2993788

[B48] Zhu Z. Ding X. Zhang D. Wang L. (2020). Weakly-supervised balanced attention network for gastric pathology image localization and classification, in 2020 IEEE 17th International Symposium on Biomedical Imaging (ISBI) (Iowa City, IA: IEEE), 1–4.

